# Supplementary material for: A set of multi-entry identification keys to African frugivorous flies (Diptera, Tephritidae)
Source: Zookeys. 2014 Jul 24;(428):97–108. doi: 10.3897/zookeys.428.7366 (PMC4143993; doi:10.3897/zookeys.428.7366)
Supplement: Supplementary material 6 — Key to Ceratitis [file zookeys-428-097-s006.zip › SF6_ZooKeys_key to Ceratitis/key/SF6_ZooKeys_key to Ceratitis/Media/Html/Ceratitis divaricata .htm]

Ceratitis (Ceratalaspis) divaricata (Munro)


***Ceratitis*** **(*Ceratalaspis*)
*divaricata* (Munro)**

*Hoplolophomyia divaricata* Munro, 1933a: 31.

Body
length: 6.32 (5.65-7.10) mm; wing length: 6.54 (6.15-6.85) mm.

Male

Head.
Antenna yellow. Third antennal segment twice as long as second segment. Arista
almost bare, extremely short hairs which are hardly visible. Frons with short
scattered hairs which are usually distinctly darker than frons, sometimes
largely the same colour; convex to flat, in lateral view not distinctly
projecting forwards at antennal implant. Chaetotaxy normal for subgenus.

Thorax.
Postpronotum white or dirty white, unspotted. Ground colour of mesonotum pale
with yellow-orange tinge; mesonotum, intra-alar spots sometimes less developed.
Chaetotaxy normal for subgenus. Scapular setae pale. Two anepisternal bristles,
lower bristle sometimes weaker and brownish coloured. Scutellum white basally,
otherwise yellow with three black separate markings apically; basally without
dark spots, but two subapical spots. Subscutellum pale with a single median spot.

Legs
yellow; setation typical for subgenus, mainly pale especially on femora.
Posterior and posterodorsal rows on fore femur pale; ventral row yellow.

Wing
bands with extensively yellow markings. Banding, two longitudinal bands,
marginal band continuous, band along vein M; cubital and medial bands not
present; crossvein r-m before middle of discal cell. Crossvein dm-cu
posteriorly more inwards than anteriorly.

Abdomen.
Yellow. Setation and banding typical for subgenus.

Female

As
male except for the following characters: Arista with slightly longer hairs,
but still very short and hardly visible. Oviscape shorter than abdominal terga
3-6 combined.

(Description
after De Meyer, 1998)
